# Chronic Inflammatory Demyelinating Polyneuropathy With Concurrent Membranous Nephropathy: An Anti-paranode and Podocyte Protein Antibody Study and Literature Survey

**DOI:** 10.3389/fneur.2018.00997

**Published:** 2018-11-27

**Authors:** Yu Hashimoto, Hidenori Ogata, Ryo Yamasaki, Takakazu Sasaguri, Senri Ko, Kenichiro Yamashita, Zhang Xu, Takuya Matsushita, Takahisa Tateishi, Shin'ichi Akiyama, Shoichi Maruyama, Akifumi Yamamoto, Jun-ichi Kira

**Affiliations:** ^1^Department of Neurology, Japan Community Health Care Organization Kyushu Hospital, Fukuoka, Japan; ^2^Department of Neurology, Neurological Institute, Graduate School of Medical Sciences, Kyushu University, Fukuoka, Japan; ^3^Department of Pathology, Japan Community Health Care Organization Kyushu Hospital, Fukuoka, Japan; ^4^Division of Nephrology, Department of Internal Medicine, Nagoya University Graduate School of Medicine, Nagoya, Japan

**Keywords:** contactin-1, autoantibody, chronic inflammatory demyelinating polyneuropathy, membranous nephropathy, nephrotic syndrome

## Abstract

**Background:** Several case reports have described the concurrence of chronic inflammatory demyelinating polyneuropathy (CIDP) and membranous nephropathy (MN). The presence of autoantibodies against podocyte antigens phospholipase A2 receptor (PLA2R) and thrombospondin type 1 domain containing 7A (THSD7A) in MN suggests an autoimmune mechanism. Some CIDP patients also harbor autoantibodies against paranodal proteins such as neurofascin 155 (NF155) and contactin-1 (CNTN1). We investigated the relationship between CIDP and MN by assaying autoantibodies against paranodal and podocyte antigens in a CIDP patient with MN, and by a literature survey on the clinical features of CIDP with MN.

**Methods:** Anti-CNTN1 and NF155 antibodies were measured by flow cytometry using HEK293 cell lines stably expressing human CNTN1 or NF155. Binding capacity of antibodies was validated by immunostaining mouse teased sciatic nerve fibers. Anti-PLA2R antibodies were measured by enzyme-linked sorbent assay and anti-THSD7A antibodies by indirect immunofluorescence assay. Clinical features between 14 CIDP with MN cases including two with anti-CNTN1 antibodies and 20 anti-CNTN1 antibody-positive CIDP cases were compared.

**Results:** A patient whose ages was in the late 70 s complained of progressive weakness and superficial and deep sensory impairment in four extremities over 6 months. Nerve conduction studies showed prominent demyelination patterns. The patient presented with nephrotic syndrome. Renal biopsy disclosed basement membrane thickening with local subepithelial projections and glomerular deposits of IgG4, compatible with MN. Autoantibody assays revealed the presence of IgG4 and IgG1 anti-CNTN1 antibodies, but an absence of anti-NF155, anti-PLA2R, and anti-THSD7A antibodies. The patient's serum stained paranodes of teased sciatic nerves. CIDP with MN and anti-CNTN1 antibody-positive CIDP commonly showed male preponderance, relatively higher age of onset, acute to subacute onset in 35–50% of cases, distal dominant sensorimotor neuropathy, proprioceptive impairment leading to sensory ataxia, and very high cerebrospinal fluid protein levels. However, 11 of 13 CIDP patients with MN had a favorable response to mono- or combined immunotherapies whereas anti-CNTN1 antibody-positive CIDP was frequently refractory to corticosteroids and intravenous immunoglobulin administration.

**Conclusion:** CIDP with MN and anti-CNTN1 antibody-positive CIDP show considerable overlap but are not identical. CIDP with MN is probably heterogeneous and some cases harbor anti-CNTN1 antibodies.

## Introduction

Chronic inflammatory demyelinating polyneuropathy (CIDP) is an acquired immune-mediated disorder affecting the peripheral nerves, classically characterized by symmetrical weakness and impaired sensation. Although it is assumed that cell-mediated and humoral immunity play roles in this disease, the cause of CIDP remains to be elucidated. Recently, autoantibodies against paranodal proteins, such as neurofascin 155 (NF155) (1, 2), contactin-1 (CNTN1) (3–5), and contactin-associated protein 1 (CASPR1) (6), were reported to be present among subsets of CIDP patients.

Several case reports have described the concurrence of CIDP and membranous nephropathy (MN) (7–16), which is a major cause of nephrotic syndrome in adults. The autoimmune nature of idiopathic MN was clearly delineated by the identification of autoantibodies against podocyte antigens, namely phospholipase A2 receptor (PLA2R) and thrombospondin type 1 domain containing 7A (THSD7A) (17). Only one CIDP case with MN has been examined for paranodal protein antibodies and was found to be positive for anti-CNTN1 antibody (5). CNTN1 belongs to the immunoglobulin superfamily and is an adhesion molecule. Axonal CNTN1 and CASPR1 form septate-like junctions together with glial NF155 to maintain ion channel clustering at nodes of Ranvier (18). Because the loss of CNTN1 in genetically modified mice leads to decreased nerve conduction velocity (19), CNTN1 is regarded as fundamental for maintaining saltatory conduction. However, the roles of anti-CNTN1 antibodies in concomitant MN are unknown. In a previous report of anti-CNTN1 antibody-positive CIDP with MN, neither anti-PLA2R nor anti-THSD7A antibodies were studied (5). Therefore, a common pathway leading to both CIDP and MN remains to be elucidated. In the present study, we aimed to clarify the relationship between CIDP with MN and anti-CNTN1 antibody-positive CIDP by searching for autoantibodies to paranodal antigens and podocyte antigens in a patient with CIDP and MN, and by comparing the clinical features of CIDP with MN and anti-CNTN1 antibody-positive CIDP.

## Materials and methods

### Case presentation

**Figure 1:**
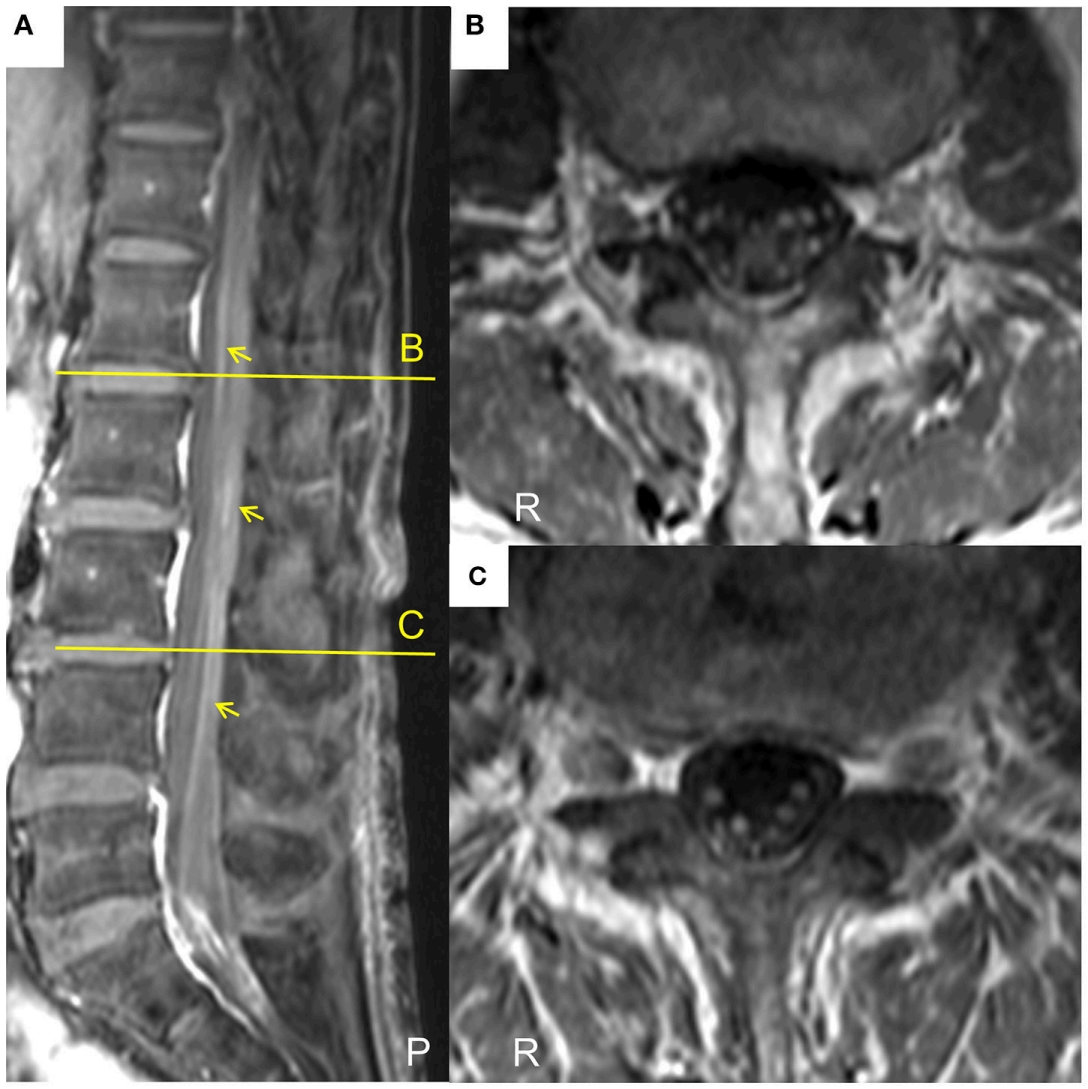
Lumbar magnetic resonance images of the current case of a CIDP patient with MN. **(A)**The sagittal T1-weighted spectral adiabatic inversion-recovery (SPAIR) magnetic resonance image shows gadolinium enhancement of cauda equine (arrows). **(B,C)** Axial T1-weighted SPAIR magnetic resonance images at the level of the indicated lines in **(A)**. Thickening and gadolinium enhancement of the cauda equine are visible. P, posterior; R, right.

A patient whose age was in the late 70 s was referred to our hospital because of progressive weakness and paresthesia of the extremities over 6 months without any prodromal episode. The patient had no medical history for any neurological, renal, or collagen diseases. Physical examination revealed oral dryness, dry skin, bilateral leg edema, and wheezing in the right lower lung field. Neurologically, the patient showed symmetrical weakness that was more marked in the legs than in the arms, generalized areflexia, and severe superficial and deep sensory impairment in the distal parts of the four extremities. As a result, the patient could not walk without help. Mild orthostatic hypotension was observed. The patient showed no tremor or cranial nerve involvement. Nerve conduction studies showed prominent demyelinating patterns in all nerves examined, such as low-amplitude compound muscle action potentials with temporal dispersion, prolonged distal latencies, and reduced motor conduction velocities (Table 1), which met the European Federation of Neurological Societies/Peripheral Nerve Society electrophysiological criteria for definite CIDP. F waves and sensory nerve action potentials were not evoked. Magnetic resonance imaging showed thickening and gadolinium enhancement of the cauda equine (Figure 1). However, no demyelinating lesions of central nervous system tissues were detected.

**Table 1:** Serial nerve conduction study findings in the present case.

	**Normal**	**The present case**	
Time from onset (month)		7	11
Treatment status		Before treatments	After treatments
Side		L/R	L/R
**MEDIAN NERVE**			
Distal latency (ms)	< 4.2	12.2/14.9	6.6/10.3
MCV (m/s)	>48	35.5/23.9	41.8/31.7
CMAP amplitude (mV)	>3.5	1.75/0.36	2.59/0.43
F wave latency (ms)	< 31	NR/NR	38.3/NR
SCV (m/s)	>44	NR/NR	NR/NR
SNAP amplitude (μV)		NR/NR	NR/NR
**ULNAR NERVE**			
Distal latency (ms)	< 3.4	7.1/7.2	4.1/5.0
MCV (m/s)	>49	33.3/21.9	31.2/39.1
CMAP amplitude (mV)	>2.8	1.10/2.99	5.47/3.62
F wave latency (ms)	< 32	NR/NR	41.3/42.1
SCV (m/s)	>44	NR/NR	NR/NR
SNAP amplitude (μV)		NR/NR	NR/NR
**TIBIAL NERVE**			
Distal latency (ms)	< 6.0	9.1/8.8	8.4/8.0
MCV (m/s)	>41	22.9/18.0	29.8/26.7
CMAP amplitude (mV)	>2.9	0.96/0.66	1.26/1.81
F wave latency (ms)	< 58	NR/NR	77.1/74.4
**SURAL NERVE**			
SCV (m/s)	>45	NR/NR	NR/NR
SNAP amplitude (μV)		NR/NR	NR/NR

*CMAP, compound muscle action potentials; MCV, motor nerve conduction velocity; L, left; NR, not recordable; R, right; SCV, sensory nerve conduction velocity; SNAP, sensory nerve action potentials*.

Regarding the cause of bilateral leg edema, the patient had severe hypoalbuminemia (0.7 g/dl), hypercholesterolemia (346 IU/l), and proteinuria (4.1 g in 24 h), which met the diagnostic criteria for nephrotic syndrome. Renal biopsy revealed stage 2 idiopathic membranous nephropathy (MN), as shown in the Results section. Other blood tests revealed positive anti-SSA/Ro antibodies (1:16), low vitamin B_12_ (57 pg/ml), and hypothyroidism (TSH 5.30 μIU/ml, FT4 0.83 ng/dl). Serological tests for infectious diseases (hepatitis B and C viruses, human immunodeficiency virus, and syphilis), anti-SSB/La antibodies, rheumatoid factor, anti-double-stranded DNA antibodies, anti-neutrophil cytoplasmic antibodies, M-protein, and cryoglobulins were negative. The patient's cerebrospinal fluid (CSF) protein level was 61 mg/dl and white blood cell count was 1/μl. There was no evidence of malignant tumor in the upper and lower gastrointestinal tracts by endoscopies and computed tomography. The patient was also diagnosed with Sjögren's syndrome based on increased anti-SSA/Ro antibodies and relevant clinical findings, such as xerostomia, xerophthalmia, lymphocytic interstitial pneumonia, atrophic gastritis, and subclinical hypothyroidism.

### Generation of transformed cell lines stably expressing human CNTN1

We generated a HEK293 cell line stably expressing human CNTN1-turbo green fluorescent protein (GFP) fusion proteins using a plasmid containing a full-length cDNA encoding human CNTN1 (OriGene Technologies Inc., Rockville, MD). The plasmids were transfected into naive HEK 293 cells using FuGENE6 (Promega Corporation, Madison, WI) according to the manufacturer's recommendations. The transfected cells were cultured in selection medium containing G418 (Thermo Fisher Scientific, Waltham, MA). When single colonies of adequate size for clonal expansion had formed, individual colonies were picked up using a sterile cloning cylinder and scaled up to larger volumes.

### Flow cytometric and conventional cell-based assay for anti-NF155 or anti-CNTN1 antibodies

Anti-CNTN1 antibodies were measured by the same methodology used for measuring anti-NF155 antibodies, as described previously (2). In brief, CNTN1-turbo GFP-transfected and naive HEK293 cells were evenly mixed and resuspended in Dulbecco's modified Eagle's medium containing 1% fetal bovine serum and 1 mM ethylenediaminetetraacetic acid (EDTA) (FCM buffer) at a concentration of 1.0 × 10^6^ cells/ml, and rotated at 4°C for 60 min. Serum samples (2.5 μl) were mixed with 47.5 μl of cell-containing solution (1:20 dilution). After incubation at 4°C for 60 min, cells were washed and bound IgG was detected with Alexa Fluor 647-labeled anti-human IgG antibodies (Thermo Fisher Scientific) diluted 1:500 with FCM buffer. After incubation at 4°C for 60 min, cells were washed, resuspended in 100 μl phosphate-buffered saline (PBS) containing 5 mM EDTA and analyzed by MACSQuant Analyzer (Miltenyi Biotec, Bergisch Gladbach, Germany). The mean fluorescence intensity (MFI) of cell-associated turbo GFP and Alexa 647 was measured for each sample. The MFI of cell-associated Alexa 647 was measured to detect human IgG bound to CNTN1, using cells without CNTN1 expression as a negative control. For each serum sample, the MFI ratio was calculated by dividing Alexa 647 MFI of CNTN1-transfected cells by Alexa 647 MFI of CNTN1-untransfected cells, and the ΔMFI was calculated by subtracting Alexa-647 MFI of CNTN1-untransfected cells from Alexa 647 MFI of CNTN1-transfected cells. As a conventional cell-based assay, cell-containing solutions were placed on glass slides and viewed under a confocal microscope (A1; Nikon, Tokyo, Japan). In an anti-CNTN1 antibody-positive patient, IgG subclass profiles were examined using phycoerythrin-conjugated mouse anti-human IgG1, IgG2, IgG3, and IgG4 antibodies (Beckman Coulter Inc., Brea, CA) at 1:500 dilution.

### Immunostaining of mouse teased sciatic nerve fibers

Sciatic nerves were obtained from C57BL/6 mice, dissected, and fixed for 10 min in freshly prepared PBS containing 4% paraformaldehyde. After washing with PBS, fixed nerves were teased and transferred onto glass slides, permeabilized with 2% Triton X-100 in PBS for 30 min, blocked in 10% goat serum and 1% Triton X-100 in PBS for 60 min, and then incubated at 4°C in blocking solution containing rabbit anti-CASPR1 antibodies (diluted 1:500) (Abcam, Cambridge, UK) and sera from anti-CNTN1 antibody-positive CIDP patients or healthy controls (HCs) (diluted 1:100). After 24 h, teased nerve fibers were washed three times in PBS for 30 min, and incubated for 60 min with Alexa 488-labeled anti-human IgG and Alexa 647-labeled anti-rabbit IgG (Thermo Fisher Scientific), diluted 1:500. Finally, teased nerve fibers were washed three times in PBS for 30 min, mounted with PermaFluor (Thermo Fisher Scientific), and examined by confocal microscopy (A1; Nikon), using 488- and 638-nm lasers for excitation.

### Assay for Anti-PLA2R or Anti-THSD7A antibodies

Anti-PLA2R antibodies were measured by a commercial ELISA kit (Euroimmun AG, Lübeck, Germany) (20) and anti-THSD7A antibodies were assayed by a commercial indirect immunofluorescence assay kit (Euroimmun AG) (21).

### Pathological and immunofluorescence studies for renal biopsy specimens

Renal biopsy was performed to investigate the cause of the nephrotic syndrome. Tissues for light microscopy and immunofluorescence were routinely fixed in formalin and embedded in paraffin. The formalin-fixed paraffin-embedded sections were cut at 2 μm and stained with hematoxylin and eosin, periodic acid-Schiff, periodic acid methenamine silver, Masson's trichrome, and Congo red. Immunofluorescence staining was performed using formalin-fixed paraffin-embedded sections that were prepared by protease K (Dako, Copenhagen, Denmark) for 60 min. Fluorescein isothiocyanate-labeled rabbit anti-human IgG, IgA, IgM, C1q, C3, fibrinogen, kappa- and lambda-light chains (Dako) were used. Additionally, mouse monoclonal anti-human IgG1, IgG2, IgG4 (Chemicon, Temecula, CA, USA), and IgG3 (Thermo Fisher Scientific) antibodies, and rabbit polyclonal anti-PLA2R antibodies (Abcam) were used followed by Alexa 546 goat anti-mouse IgG (H+L) and Alexa 488 goat anti-rabbit IgG (H+L) (Thermo Fisher Scientific).

### Literature reviews

A PubMed search was performed to identify previous reports of CIDP with concurrent MN, using “CIDP” and “membranous nephropathy” or “membranous glomerulonephritis” as keywords. We reviewed all suitable cases by literature survey.

## Results

### Autoantibody studies for nodal and podocyte proteins

**Figure 2:**
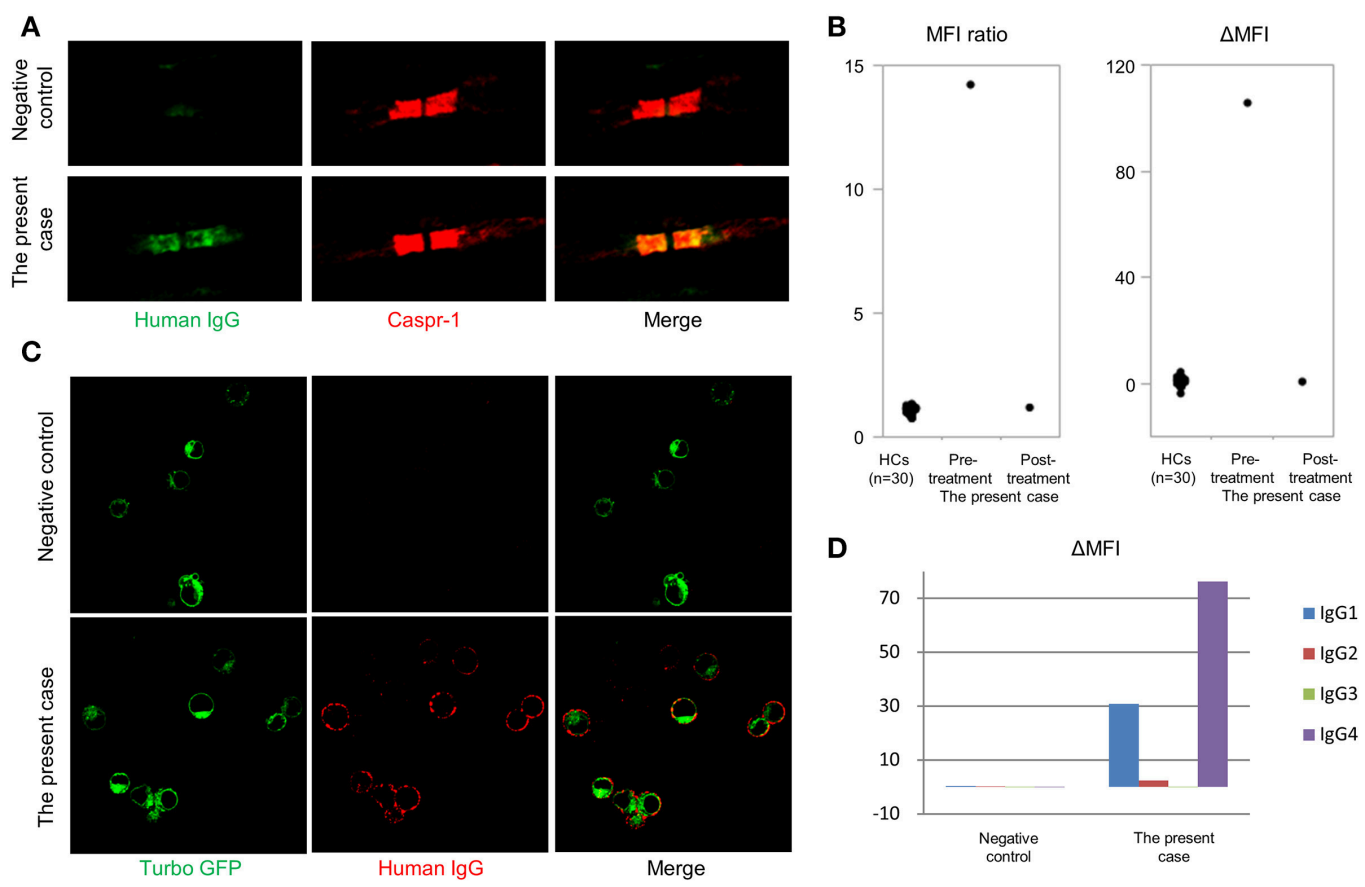
Detection of anti-contactin-1 antibodies. **(A)** Double immunostaining of mouse teased sciatic nerve fibers with anti-contactin-associated protein 1 (CASPR1) antibodies and sera from the anti-CNTN1 antibody-positive CIDP case or an anti-CNTN1 antibody-negative HC. A similar paranodal staining pattern is observed for anti-CASPR1 antibodies and the patient's serum. Scale bar = 5 μm. **(B)** Mean fluorescence intensity (MFI) ratio and ΔMFI for anti-contactin-1 (CNTN1) antibodies. Serum from the present case at admission (6th months from the onset) shows significantly higher MFI ratio and ΔMFI values (14.2 and 106, respectively) than those of 30 HCs (1.1 and 0.47, respectively). Standard deviation of MFI ratio and ΔMFI are 0.16 and 1.43, respectively. Eleven months after starting the treatment (18th months from the onset), anti-CNTN1 antibodies in the patient's serum disappeared concurrent with clinical and electrophysiological improvement. **(C)** Conventional cell-based assay shows that IgG in the patient's serum binds to HEK293 cells stably expressing human CNTN1-turbo green fluorescent protein (GFP). Scale bar = 20 μm. **(D)** Subclass analysis of anti-CNTN1 antibodies from the present case reveal the predominance of IgG1 and IgG4 subclasses.

Autoantibodies in the patient's serum bound to the paranodal regions of mouse teased sciatic nerve fibers, with a staining pattern similar to anti-CASPR1 antibody (Figure 2A). Flow cytometry for anti-CNTN1 antibodies demonstrated the present case had a higher MFI ratio and ΔMFI values (the MFI ratio and ΔMFI were 14.2 and 106, respectively) than those of 30 HCs (mean MFI ratio and ΔMFI were 1.1 and 0.5, respectively; standard deviation of MFI ratio and ΔMFI were 0.16 and 1.43, respectively) (Figure 2B). A conventional cell-based assay visualized IgG binding to CNTN1-expressing HEK293 cells (Figure 2C). Subclass analysis of anti-CNTN1 antibodies revealed a predominant increase in IgG4 and IgG1 (Figure 2D). The MFI ratio and ΔMFI values of anti-NF155 antibodies were 1.04 and 1.02, respectively, and were similar to those of 10 healthy controls (mean MFI ratio and ΔMFI were 0.92 and −1.6, respectively; standard deviation of MFI ratio and ΔMFI were 0.14 and 4.0, respectively). However, for podocyte antigens, anti-PLA2R antibodies and anti-THSD7A antibodies were not positive in the serum. Finally, the patient was diagnosed with anti-CNTN1 antibody-positive CIDP with concurrent MN.

### Histological and immunofluorescence studies of biopsied renal tissue

**Figure 3:**
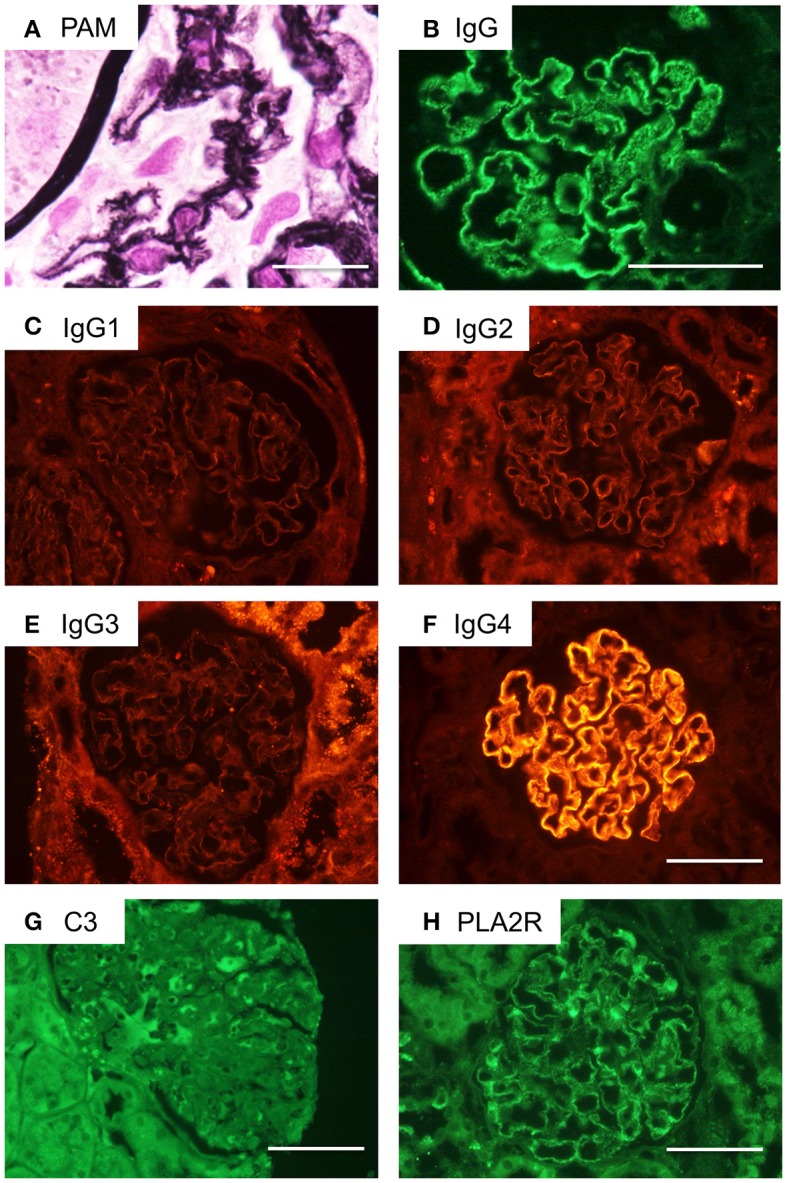
Renal pathological findings in the current case of a CIDP patient with MN. **(A)** Periodic acid methenamine silver (PAM) staining of the patient's glomeruli reveals a spiked appearance of the glomerular basement membrane. Scale bar = 50 μm. **(B)** Immunofluorescence microscopy demonstrates granular deposits of IgG along the glomerular basement membrane. Scale bar = 100 μm. **(C–F)** IgG subclass staining indicated only IgG4 deposits were detectable. Scale bar = 100 μm. **(G)** C3 deposition is not obvious by immunohistochemistry. Scale bar = 100 μm. **(H)** Immunostaining for phospholipase A2 receptor (PLA2R) is weakly positive. Scale bar = 100 μm.

The glomeruli showed diffuse spikes of the glomerular basement membrane by periodic acid methenamine silver staining (Figure 3A). Immunofluorescence microscopy demonstrated granular deposits of IgG along the glomerular basement membrane (Figure 3B). These findings were consistent with stage 2 idiopathic MN. In an IgG subclass study, only IgG4 was detectable (Figures 3C–F). Deposition of complement C3 was not conspicuous. Staining for PLA2R was weakly positive by immunofluorescence (Figures 3G,H).

### Correlation of Anti-CNTN1 antibody levels with clinical course

Seven months after disease onset, the patient was treated with methylprednisolone pulse therapy (1000 mg/day for 3 days) followed by oral prednisolone (1 mg/kg/day with gradual taper). However, the patient had little improvement in muscle weakness and sensory disturbance, even though NCS findings tended to improve and the serum albumin level was increased (2.5 g/dl). Two months after initial treatment, intravenous immunoglobulin (IVIg) (400 mg/kg/day for 5 days) was administered. Thereafter, the patient's sensorimotor disturbance was ameliorated. Two months after the additional treatment, the patient was able to walk without help and NCS findings continued to improve (Table 1). Eleven months after starting immunotherapies (18th months from disease onset), the patient's anti-CNTN1 antibodies were undetectable (MFI ratio and ΔMFI were 1.40 and 0.97, respectively).

### Characteristic features of CIDP with concurrent MN and comparison with Anti-CNTN1 antibody-positive CIDP

By literature survey, we found 13 previously reported cases of CIDP and MN. The summary of 14 cases including the current case is shown in Table 2. Ten cases preceded CIDP while four showed concurrent onset of CIDP and MN. The mean age at the onset of CIDP was 47.6 ± 21.6 years (range 9–81 years old; 36% >60 years). The male-to-female ratio was 10:4 (2.5:1). Chronic onset of CIDP was seen in nine cases while four cases had acute onset ( ≤ 1 month) and one showed subacute onset (6 weeks). All patients were regarded as typical CIDP. Eleven cases (79%) showed distal dominant symmetrical sensory impairment while proprioception impairment or sensory ataxia was described in at least eight cases (57%). All but two cases had very high CSF levels (mean 291 ± 330 mg/dl; range 61–1,320 mg/dl; 12 cases (86%) >100 mg/dl).

**Table 2:** CIDP with concurrent membranous nephropathy: A literature review.

**References**	**Age at onset of CIDP (years)/gender**	**Period from onset to peak of CIDP**	**Motor functions at peak of illness**
Witte et al. (7)	43/M	>2 months	Ataxic gait, mild limb weakness
Kohli et al. (8)	18/M	>2 months	A steppage ataxic gait
Panjwani et al. (9)	55/M	4 weeks	Mild weakness in four extremities (MRC scale: grade 4)
Kanemoto et al. (10)	9/M	Acute-like GBS	Running disturbance
Mobbs et al. (11)	81/F	>2 months	Multiple falls due to weakness in the legs
Wu et al. (12)	53/F	>2 months	Symmetric weakness of the limbs affecting distal muscles (MRC scale: grade 3) more than proximal muscles (MRC scale: grade 4)
Wu et al. (12)	62/M	>2 months	Unsteady gait
Emsley et al. (13)	66/M	6 weeks	Tetraparesis most marked distally (MRC scale: grade 2-3)
Chen et al. (14)	60/M	>2 months	Unsteady gait and clubby movement of four extremities
Smyth et al. (15)	25/M	>2 months	Mild weakness (MRC scale: grade 4)
Wong et al. (16)	36/M	<1 month	Unable to walk
Wong et al. (16)	33/F	>2 months	Unsteady gait
Doppler et al. (5)	48/M	Acute-like GBS	Tetraparesis
The present case	78/F	>2 months	Unable to walk

**Table:** 

**References**	**Age at onset of CIDP (years)/gender**	**CIDP subtype**	**Sequence of manifestations**	**Sensory ataxia or disturbed deep sensation**	**CSF protein levels (mg/dl)**	**Anti-CNTN1 antibodies**	**Serum albumin (mg/dl)**
Witte et al. (7)	43/M	Typical	Neuro → renal	Romberg sign (+)	280	ND	ND
Kohli et al. (8)	18/M	Typical	Neuro → renal	Sensory ataxia	64	ND	ND
Panjwani et al. (9)	55/M	Typical	Neuro → renal	Decreased proprioception	145	ND	4.0
Kanemoto et al. (10)	9/M	Typical	Neuro → renal	Sensory ataxia	212	ND	3.1
Mobbs et al. (11)	81/F	Typical	Neuro → renal	Decreased proprioception	120	ND	2.9
Wu et al. (12)	53/F	Typical	Neuro → renal	Vibratory loss	1320	ND	3.2
Wu et al. (12)	62/M	Typical	Neuro → renal	Vibratory loss	129	ND	2.1
Emsley et al. (13)	66/M	Typical	Concurrent	Decreased proprioception	185	ND	2.4
Chen et al. (14)	60/M	Typical	Neuro → renal	ND (sensory disturbance)	299	ND	2.2
Smyth et al. (15)	25/M	Typical	Neuro → renal	Decreased vibratory sensation	316	ND	3.7
Wong et al. (16)	36/M	Typical	Neuro → renal	ND (severe sensory disturbance)	635	ND	ND
Wong et al. (16)	33/F	Typical	Concurrent	Sensory ataxia	102	ND	ND
Doppler et al. (5)	48/M	Typical	Concurrent	ND (severe sensory disturbance)	204	Positive	ND
The present case	78/F	Typical	Concurrent	Sensory ataxia	61	Positive	0.7

**Table:** 

**References**	**Age at onset of CIDP (years) /gender**	**Treatment and response of CIDP**	**Treatment and response of MN**
Witte et al. (7)	43/M	CS, ineffective; PE, improved	CS, ineffective; PE, ineffective; chlorambucil, ineffective
Kohli et al. (8)	18/M	CS, improved	CS, ineffective
Panjwani et al. (9)	55/M	CS, improved	CS, ineffective; IVIg, ineffective
Kanemoto et al. (10)	9/M	CS, improved	CS, improved
Mobbs et al. (11)	81/F	PE+CS+AZT, mild improvement	ND
Wu et al. (12)	53/F	IVIg+CS+PE, no improvement other than tremor	IVIg+CS+PE, ineffective
Wu et al. (12)	62/M	PE, improved; CS, further improvement	PE+CS, improved
Emsley et al. (13)	66/M	Spontaneously improved	ACE inhibitor, improved
Chen et al. (14)	60/M	CS, improved; cyclophosphamide, further improved	CS+cyclophosphamide, improved
Smyth et al. (15)	25/M	PE+MTX, improved	PE+MTX, improved
Wong et al. (16)	36/M	IVIg+CS+PE+cyclosporin or tacrolimus, no improvement	IVIg+CS+PE+cyclosporin or tacrolimus, no improvement
Wong et al. (16)	33/F	CS, improved	CS+cyclophosphamide, improved
Doppler et al. (5)	48/M	IVIg, initial improvement; CS, transiently improved; PE, improved	Complete recovery (treatment efficacy was not well documented)
The present case	78/F	CS, ineffective; IVIg, improved	CS, improved

*ACE, angiotensin converting enzyme; AZT, azathioprine; CS, corticosteroids (oral high dose or methylprednisolone pulse therapy); CIDP, chronic inflammatory demyelinating polyneuropathy; CNTN1, contactin-1; CSF, cerebrospinal fluid; F, female; GBS, Guillain-Barré syndrome; IVIg, intravenous immunoglobulin; M, male; MRC, Medical Research Council; MTX, methotrexate; ND, not documented; PE, plasma exchange*.

Regarding the efficacy of immunotherapies on CIDP, corticosteroids were effective in 5/7 (71.4%) cases with monotherapy. Plasma exchange (PE) was effective in 3/3 (100%) cases with monotherapy. IVIg was effective in 2/2 (100%) cases with monotherapy. Combined immunotherapies were performed in four cases (one each of PE + methotrexate, PE + CS + azathioprine, PE + CS + IVIg, PE + CS + IVIg + cyclosporine), and were effective only in one case (PE + methotrexate). These observations suggest that most cases (11, 79%) responded favorably to immunotherapies such as CS, PE, and IVIg initially; however, three cases (21%) were refractory even to combined treatments including three or more immunotherapies.

Regarding the efficacy of immunotherapies on MN, favorable responses were described in 2/5 (40%) cases by CS monotherapy, in 0/1 (0%) by PE monotherapy, in 0/1 (0%) by IVIg monotherapy, and in 0/1 (0%) by immunosuppressants. Combined immunotherapies, including CS + immunosuppressant, CS + PE, PE + immunosuppressant, PE + CS + IVIg, and PE + CS + IVIg + immunosuppressant, were effective in 4/6 (67%) cases. Overall, only two cases responded favorably to monotherapy and five required combined immunotherapies to achieve improvement. There were at least five cases refractory to mono- or combined immunotherapies.

By literature survey, three case series reported 20 anti-CNTN1 antibody-positive CIDP patients (3, 4, 5) (Table 3). Ages at onset were 63.0 ± 13.5 (mean ± SD) years (range 33–81 years; 80% >60 years). The male-to-female ratio was 14:6 (2.3:1). Ten had a chronic progressive course while seven showed an acute or rapidly progressive course and three had subacute onset. Common clinical features were symmetrical manifestation, distal dominant involvement (90%) especially in sensory impairment, predominant sensory ataxia, an aggressive disease course, and poor response to CS and IVIg. Their CSF protein level was 253 ± 143 (mean ± SD) mg/dl (range: 79–693 mg/dl; 95% >100 mg/dl). Thus, common features between CIDP with MN and anti-CNTN1 antibody-positive CIDP are as follows (Table 3): male preponderance (about 2.5-fold higher than female), occasional acute to subacute onset (35.7 and 50%), relatively higher age of onset, distal dominant sensorimotor neuropathy with frequent proprioceptive sensory impairment leading to sensory ataxia, and high CSF protein levels. However, CIDP with MN was occasionally seen in younger people (< 30 years) and had a more favorable response to immunotherapies compared with anti-CNTN1 antibody-positive CIDP.

**Table 3:** Comparison of clinical features between CIDP with MN and anti-CNTN1 antibody-positive CIDP.

**Feature**	**CIDP with MN (*n* = 14)**	**Anti-CNTN1 antibody-positive CIDP (*n* = 20)**
Male to female ratio	10:4 (2.5:1)	14:6 (2.3:1)
Age at onset of CIDP (mean ± SD, years)	47.6 ± 21.6 (range 9–81)	63.0 ± 13.5 (range 33–81)
Onset age of CIDP > 60 years	5 (36%)	16 (80%)
**MODE OF ONSET**		
Acute onset	4	7
Subacute onset	1	3
Chronic onset	9	10
Sensorimotor neuropathy	11 (79%)*	19 (95%)
Distal dominant muscle weakness	11 (79%)	14 (70%)
Proprioceptive impairment or sensory ataxia	8 (57%)	15 (75%)
CSF protein amounts (mean ± SD, mg/dl)	291 ± 330 (range 61–1320)	253 ± 143 (range 79-693)
CSF protein > 100 mg/dl	12 (86%)	19 (95%)
**EFFICACY OF IMMUNOTHERAPIES ON CIDP**		
CS	5/7 (71%)	5/17 (29%)***
PE	3/3 (100%)	5/7 (71%)
IVIg	2/2 (100%)	4/7 (57%)****
Combined	1/4 (25%)**	ND

CIDP with MN cases are derived from Witte et al. (7), Kohli et al. (8), Panjwani et al. (9), Kanemoto et al. (10), Mobbs et al. (11), Wu et al. (12), Emsley et al. (13), Chen et al. (14), Smyth et al. (15), and Wong et al. (16) and the present case and other anti-CNTN1 antibody-positive CIDP cases are from Querol et al. (3), Doppler et al. (4), and Miura et al. (5).

* *Cases showing only vibration sense impairment are not counted*.

** *Combined immunotherapies include one each of PE + methotrexate, PE + CS + azathioprine, PE + CS + IVIg, PE + CS + IVIg + cyclosporin*.

*** *Partial or transient response is regarded as ineffective*.

**** *Initial improvement in acute onset cases is counted as effective. CS, corticosteroids; IVIg, intravenous immunoglobulin; ND, not described; PE, plasma exchange; SD, standard deviation*.

## Discussion

The patient in this study is the second reported case of anti-CNTN1 antibody-positive CIDP with MN. Interestingly, the patient was negative for anti-PLA2R and anti-THSD7A antibodies, although IgG4 was deposited on the glomerular basement membrane in the kidney. The patient was concurrently diagnosed with Sjögren's syndrome. However, this usually manifests as chronic sensorimotor axonal polyradiculoneuropathy but not demyelinating neuropathy, and rarely accompanies MN (22, 23). Therefore, we consider anti-CNTN1 antibodies but not Sjögren's syndrome contributory to the present illness of our patient. In MN, the frequency of anti-PLA2R and anti-THSD7A antibodies are 50–80%, and 5–10%, respectively (17). Thus, target antigens in MN are still undetermined in 10–20% of cases. Our patients clearly had IgG4 deposition in the glomerular basement membrane, suggesting immune-mediated podocyte damage. However, anti-PLA2R and anti-THSD7A antibodies were double seronegative in this study patient. We could not totally exclude the involvement of low-titer anti-PLA2R antibodies because PLA2R glomerular deposits, one of the features of anti-PLA2R antibody-positive MN (24), were observed in this patient. It is worth searching for undetermined renal target antigens for CIDP with MN in the future, including CNTN1 whose mRNA is also weakly expressed in the kidney (25).

The decrease of anti-CNTN1 antibody levels along with improved CIDP by immunotherapy and staining of the paranodes by the patient's sera suggest a pathogenic role of anti-CNTN1 antibodies. Anti-CNTN1 antibodies in our patient were predominantly IgG4 and IgG1. In CIDP with anti-CNTN1 antibodies, it was reported that IgG1 and IgG3 subclasses were prominent in the acute stage of acute onset cases whereas IgG4 predominated at the chronic stage of acute onset cases as well as chronic onset cases (4, 5). IgG1 or IgG3 anti-CNTN1 antibodies may cause acute inflammation via complement activation at the acute inflammatory stage. By contrast, IgG4 does not bind to C1q or activate the classical complement pathway. Thus, blocking interactions between the CNTN1/CASPR1 complex and NF155 might be responsible for the pathogenic function of IgG4 anti-CNTN1 antibodies. Indeed, in human biopsied sural nerves, the detachment of Schwann cell terminal loops from axonal membranes at paranodes without inflammatory cell infiltration was observed by electron microscopy (26). Furthermore, IgG4 but not IgG1 can pass the paranodal barrier and disrupt the paranodal structure upon the passive transfer of anti-CNTN1 antibodies to rats (27). All these observations suggest that IgG4 anti-CNTN1 antibodies play pathogenic roles, especially in the chronic phase, by disrupting axo-glial interactions at the paranodes.

Among 14 cases of CIDP and MN found by literature survey (7–16), only two cases (including the current case) were examined for autoantibodies to paranodal antigens and both cases were positive for anti-CNTN1 antibodies (5). A comparison of the clinical features between CIDP with MN and anti-CNTN1 antibody-positive CIDP revealed male preponderance, relatively higher age of onset, occasional acute/subacute onset, distal dominant sensorimotor neuropathy suggestive of typical CIDP, frequent proprioceptive sensory impairment leading to sensory ataxia, and very high CSF protein levels as common features, although in some CIDP patients with MN including our case, CSF protein levels were not very high, possibly reflecting hypoalbuminemia caused by nephrotic syndrome. There were some differences between both conditions. None of the anti-CNTN1 antibody-positive CIDP had < 30 years of onset while three of 14 CIDP cases with MN (21.4%) were younger than 30 years at onset. Eleven of 13 treated CIDP patients with MN had a favorable response to mono- or combined immunotherapies such as CS, PE, and IVIg whereas anti-CNTN1 antibody-positive CIDP was frequently refractory to IVIg and CS. These observations suggest that CIDP with MN and anti-CNTN1 antibody-positive CIDP show some overlap but are not identical. Probably CIDP with MN is heterogeneous and some cases might be anti-CNTN1 antibody-positive CIDP. Thus, anti-CNTN1 antibodies should be surveyed in patients presenting with CIDP and MN.

## Concluding remarks

We describe the results of a detailed autoantibody study in a patient with anti-CNTN1 antibody-positive CIDP and MN, together with a comparison of the clinical features between CIDP with MN and anti-CNTN1 antibody-positive CIDP. We believe that the present study expands our understanding of the disease area related to anti-CNTN1 antibodies and that anti-CNTN1 antibodies are worth studying in cases with CIDP and MN.

## Ethics statement

The study was approved by the Kyushu University Hospital ethical standards committee.

## Author contributions

YH, HO, RY, TS, SK, KY, and JK made the study plan, conducted the research, and wrote the paper. HO, ZX, SA, and SM studied the autoantibodies. TM, TT, and AY collected patient samples, clinical data and supervised the analyses.

### Conflict of interest statement

The authors declare that the research was conducted in the absence of any commercial or financial relationships that could be construed as a potential conflict of interest.

## Abbreviations

CASPR1: contactin-associated protein 1
CIDP: chronic inflammatory demyelinating polyneuropathy
CNTN1: contactin-1
EDTA: ethylenediaminetetraacetic acid
GFP: green fluorescent protein
IVIg: intravenous immunoglobulin
MFI: mean fluorescence intensity
MN: Membranous nephropathy
PBS: phosphate-buffered saline
PLA2R: phospholipase A2 receptor
THSD7A: thrombospondin type 1 domain containing 7A.
